# Patient Telemedicine Perceptions During the COVID-19 Pandemic Within a Multi-State Medical Institution: Qualitative Study

**DOI:** 10.2196/37012

**Published:** 2022-05-13

**Authors:** Pravesh Sharma, Anthony R Sinicrope, Pamela Sinicrope, Tabetha A Brockman, Nicole M Reinicke, Ian W West, Liana M Wiepert, Amy E Glasgow, Lindsey R Sangaralingham, Ashley L Holland, Christi A Patten

**Affiliations:** 1 Behavioral Health Research Program Pyschology Mayo Clinic Eau Claire, WI United States; 2 Department of Psychiatry and Psychology Mayo Clinic Health System Eau Claire, WI United States; 3 Kern Center for the Science of Health Care Delivery Mayo Clinic Rochester, MN United States; 4 Department of Health Sciences Research Mayo Clinic Rochester, MN United States

**Keywords:** COVID-19, telehealth, video appointment, telemedicine, qualitative, pandemic, outpatient, clinical care, virtual health, patient perspective, healthcare, clinical practice

## Abstract

**Background:**

During the COVID-19 pandemic, to prevent the spread of the virus, federal regulatory barriers around telemedicine were lifted, and health care institutions encouraged patients to use telemedicine, including video appointments. Many patients, however, still chose face-2-face (f2f) appointments for nonemergent clinical care.

**Objective:**

We explored patients’ personal and environmental barriers to the use of video appointments from April 2020 to December 2020.

**Methods:**

We conducted qualitative telephone interviews of Mayo Clinic patients who attended f2f appointments at the Mayo Clinic from April 2020 to December 2020 but did not utilize Mayo Clinic video appointment services during that time frame.

**Results:**

We found that, although most patients were concerned about preventing COVID-19 transmission, they trusted Mayo Clinic to keep them safe when attending f2f appointments. Many expressed that a video appointment made it difficult to establish rapport with their providers. Other common barriers to video appointments were perceived therapeutic benefits of f2f appointments, low digital literacy, and concerns about privacy and security.

**Conclusions:**

Our study provides an in-depth investigation into barriers to engaging in video appointments for nonemergent clinical care in the context of the COVID-19 pandemic. Our findings corroborate many barriers prevalent in the prepandemic literature and suggest that rapport barriers need to be analyzed and problem-solved at a granular level.

## Introduction

Telemedicine consists of using technology and telecommunication infrastructure to deliver health care–related services remotely. Options for telemedicine include speaking to a health care provider live over a phone or video call and sending and receiving messages using a secure online messaging platform (eg, a patient portal) [1]. Before the COVID-19 pandemic, use of telemedicine and video appointments, in particular, was minimal. At the onset of the pandemic, however, regulatory barriers limiting reimbursement for telemedicine were lifted [2] to increase consumer and provider willingness to use telehealth options that would have the potential to improve patients’ access to care, reduce demand for overburdened emergency health services, limit potential disease exposure, and minimize the need for scarce personal protective equipment [3]. Despite efforts by health care systems to promote telemedicine during the pandemic, many patients still chose face-to-face (f2f) appointments for nonemergent and noninterventional clinical care [4]. This qualitative study examined patients’ perceived and environmental barriers to the use of telemedicine during the COVID-19 pandemic from April 2020 to December 2020.

A systematic review conducted before the pandemic indicated that non-English speaking patients living in low socioeconomic status neighborhoods were significantly less likely to choose a video visit. Other barriers to adopting telemedicine found that top barriers included lack of reimbursement by insurance companies, patient reluctance to engage with technology, low levels of digital literacy, and a lack of access to internet and digital devices [5]. Although insurance companies and the federal government mitigated the lack of insurance coverage for video visits shortly after the pandemic began [6], other personal- (eg, age, gender, digital skills, and knowledge) and environmental-level (eg, where the person lives, internet access, support, household size, set-up) barriers during the pandemic require investigation. We focused on personal and environmental factors that might have affected patients’ willingness or ability to use telemedicine, opting to conduct a qualitative study because little is known about how the pandemic may have shifted patients’ perceptions of telemedicine. Our results will inform strategies to support the future use of telemedicine.

## Methods

### Participants

We used a stratified, purposeful sampling strategy [7] to identify participants using the Mayo Clinic electronic health record. The eligibility criteria were (1) age greater than 18 years as of April 2020; (2) established patient at Mayo Clinic Midwest (Rochester or Mayo Clinic Health System), Florida, or Arizona; and (3) attended f2f appointments at the Mayo Clinic but did not utilize Mayo Clinic video appointment services from April 2020 to December 2020. Our goal was to include an equal number of participants by race, sex, location (Midwest, Florida, and Arizona), and outpatient primary care practice and psychiatry. We chose to focus on both primary care and psychiatry as they are settings where patients visit for health maintenance monitoring and medical management that are nonemergent, are noninvasive, and include a broad number of medical and psychiatric conditions, therefore making them suitable for a video appointment. We were especially interested in psychiatry patients and those seeking mental health care due to mounting evidence showing that anxiety and stress have increased significantly among these patients during the COVID-19 pandemic [8]. We estimated we would need about 36 interviews (12 interviews per Mayo Clinic enterprise location) to achieve data saturation whereby no new themes are being identified [9-11]. Furthermore, the goal of our purposeful sample was to glean rich in-depth information from individual interviews, and we plan to use our results to design and subsequently administer a quantitative survey assessing barriers [12]. To assess saturation, we reviewed interviews completed on an ongoing basis to assess if we were capturing an adequate range of patient experiences with our interviews.

Participants’ resident zip code was used to determine rurality based on Rural-Urban Commuting Area (RUCA) codes. For this study, we defined rural as RUCA 4-10 (vs urban if RUCA=1-3) [13]. Zip code data also allowed us to use the 2015 American Community Survey to estimate median household income as a surrogate marker for participants’ socioeconomic status [14,15].

### Study Procedure

Eligible patients were recruited via email, the Mayo Clinic Patient Portal, telephone, and mail. Social media ads were also posted on Mayo Clinic’s Facebook and Twitter accounts so patients could call to participate in the study. Patients who responded were enrolled in the study after being screened via phone and deemed eligible. Patient recruitment ended when we met the target sample size of 36 participants. Once consent was obtained, participants completed semistructured interviews via telephone, and interviews were digitally transcribed. Transcriptions were then verified for accuracy by 2 study team members (NMR, IWW). After completing the interview, participants were provided with a US $25 remuneration in the form of a cash card. Interviews were conducted between July 2021 and September 2021.

### Ethical Approval

This study was approved by the Mayo Clinic institutional review board (21-00452).

### Semistructured Interviews

We developed a semistructured interview guide that was pretested with volunteers (nurses, providers, and study staff) for duration, flow, and content. The interview guide was developed by the authors (PS, PS, TAB, CAP) and sought to elicit the following domains based on our study objectives: (1) COVID-19 experience that shaped the decision to engage in f2f appointments, (2) perceived benefits of f2f appointment and barriers to video visits, and (3) recommendations to increase future use of video appointments (see Multimedia Appendix 1). Interviews lasted approximately 45 to 60 minutes and were conducted in English by 3 study team members (NMR, IWW, and LMW). The interviews were highly flexible with probes for elaboration and clarification to obtain detailed accounts. This type of interview is advantageous when limited knowledge exists about the phenomena of interest [10,11].

### Qualitative Analysis

QSR NVivo software, version 10 [16], helped facilitate response-theme generation with codes and categories based on themes emerging from the interviews. Two study team members (PS2 and ARS) coded responses together, discussing any coding discrepancies until reaching consensus and consulting a third study member (PS1) when necessary [17]. We extracted themes for analysis with code endorsement or elaboration in several interviews. In addition to open coding, we conducted planned comparisons by sex, age, and clinic site.

## Results

### Participant Characteristics

The final study sample was composed of 36 participants who attended f2f appointments during the April 2020 to December 2020 period of the pandemic. The median age of participants was 60 (SD 14.31) years. Of the 36 participants, 12 resided in the Midwest, 12 resided in FL, and 12 resided in AZ. Our sample of participants was diverse in gender (16 women and 20 men), race (21 White, 11 Black, 2 biracial, and 2 Asian), and education (15 with a bachelor degree, 13 with a graduate degree, 3 with some college degrees, 2 with associate degrees, and 1 with some school). All the participants had English as their preferred language. Most participants (32/36, 89%) were urban residents, and the estimated median household income was US $75,417 (IQR $61,730-$91,045).

### COVID-19 Experiences That Shaped the Decision to Engage in F2F Appointments

We found that nearly all participants adhered to some degree of COVID-19 safety precautions. For example, 1 participant stated the following:

Well, the only thing is, we just were following the rules of wearing facemasks and not dealing with crowds and things like that. That was the change, but other than that. We got our shots as quickly as we could during the time. We would continuing go shopping and things like that but using the precautions of six-foot distances and different—whatever was recommended at that time.

Only 2 participants expressed skepticism about the pandemic: one concerning the severity of COVID-19 infections and the other doubting the information related to COVID-19 through authorities and news media. For example, a White, male participant with some college credit mentioned:

I did get COVID personally. I didn't feel that it was that bad. It was no worse than any other illness that I've had before like getting the flu...I guess I don't feel like that the illness affected me in any way, shape, or form, or anything that I know. I don't really believe that it's as serious as the media or anybody makes it out to be. I personally had it. My entire family had it. I know dozens of people that have had it, and no one even went to the doctor for it.

Nearly all participants expressed that they felt safe from COVID-19 infection when attending f2f appointments. However, a few participants stated they would prefer a video appointment if infection rates were high in their area.

All participants conveyed a general trust in the Mayo Clinic, implying that the institutional guidelines limiting the number of persons in the patient waiting rooms and the patient screening of COVID-19 symptoms entering the hospital made them feel safe attending f2f appointments. Participants emphasized that personally adhering to masking and social distancing guidelines also made them feel safe attending in-person appointments at the Mayo Clinic. For example, a White, male participant with a bachelor’s degree stated:

I never thought about delaying care. I always felt safe coming to the clinic. I think about it even then when Mayo required masks. It’s like, well, yeah, it relieves you a little bit...Well, if we need to go, well, we’ll go. We felt safe. I think if I see the rate—if the rates went [up] or it’s been getting bad, I would prefer to use a virtual appointment if I could.

### Patient Perceived Barriers to Video Appointments

We identified 9 major themes within this domain that are reported subsequently: (1) f2f rapport, (2) f2f diagnostic and therapeutic advantages, (3) habit, (4) privacy and internet security, (5) digital literacy, (6) internet access, (7) bodily intimacy, (8) billing, and (9) patient portal. The corresponding quotes related to themes can be found in Table 1.

**Table 1:** Overview of thematic barriers to video appointments, with representative quotes.

Barrier and subdivision			Representative quotes from participants
**Face-to-face rapport**			
	Personability	“Just the comfort level. If we just met with somebody, it’s so much easier to explain.” [57-year-old Asian man, Midwest] “I can express myself more, maybe, with her [provider], with her [provider] questions and answers and whatever when I’m there.” [82-year-old White woman, AZ] “I think video visits are just not personal enough. They can see maybe a hundred people on video a day.” [60-year-old African American man, AZ] “You just have a little screen to look at…I’m old fashioned I suppose.” [57-year-old White man, Midwest]	
	Visual emphasis	“...I’m a visual person. I have to see for talking to—I prefer that rather than doing—I know it would have been too, but it’s just not the same as things upfront and person to me.” [70-year-old African American woman, AZ] “I just like to look in the eyes and be there when the provider’s talking to me versus doing it on the video.” [72-year-old White man, FL] “Actually, seeing the person.” [67-year-old African American man, FL]	
	Auditory emphasis	“When I want to go see the doctor, I want to speak to my doctor.” [30-year-old White man, Midwest] “When I go for a visit to my doctor, I like to speak to my doctor, I’m old. I’m not into all these texting people, phoning people. I like to talk to people. That gets the true picture of who they are when you sit and talk to them.” [60-year-old African American man, AZ]	
	Therapeutic emphasis	“I like my doctor a lot, and I just thought I’d feel better if I could be there and visit with her [provider], especially when I started getting depressed.” [82-year-old White woman, AZ] “It’s the physical medicine doctor, and we’re about seven years apart in age. I’ve known her for perhaps five years, and the two of us just click, and I talk to her [provider] on the phone sometimes, but seeing the actual person, your friend, your doctor, that tends to be a comfort to me.” [40-year-old White woman, Midwest] “I wanted to see her [provider] in person because also, then I could briefly talk to her about more personal things just for briefly, not for a mental health visit, but in a way it was a tiny mental health visit too.” [73-year-old White woman, AZ]	
Face-to-face therapeutic advantages			“I believe when you’re looking at somebody from a distance of feet, your understanding of basic condition is much better than across a screen. Simple things like ADHD patients, I was talking to my cousin who’s a psychiatrist, he thinks he thinks just the way the person sits, or moves, or fidgets, and the things like that you get a lot of—an experienced doctor can make out those things that are unwritten. You miss those signals when you’re across the screen.” [57-year-old Asian man, Midwest] “I’ve been to appointments with just a regular routine physical where my doctor’s seen something that I didn’t see.” [30-year-old White man, Midwest] “For some of my appointments, I think they’re okay, but when somebody needs to really check out what’s going on inside, I think a person-to-person is much better.” [61-year-old White man, FL] “The other thing is, given that this was an orthopedic complaint, I felt that there would probably be limitations to what could be observed, diagnosed, etcetera over a zoom call, and in fact, I ended up being fitted with a brace there for a few weeks during that appointment, which would have been offered if it was something.” [36-year-old White man, Midwest]
Habit			“Yeah, because I was going to see a psychiatrist...I would say it was probably just out of routine.” [50-year-old African American man, Midwest] “Other than just being new to me—I’ve never done it that way, so I guess that initial change to doing it with that method. Yeah, other than just being new to me, I’d be open to it.” [48-year-old White man, Midwest]
Privacy and security			“...when you’re on a computer, you have no idea who could be listening or who it could be—who could hear you or see your or whatever. There’s a security risk there too, and not on the hospital’s end, but on your personal computer’s end or phone or whatever. There’s always a slight security risk.” [30-year-old White man, Midwest] “I know that I would feel very, very private with it the other way. I’m not sure who would be listening to me otherwise.” [76-year-old White woman, FL] “I think that’s a wonderful platform for it, if we’re staying on top of the security, the information security, but there’s enough times that you just got to have that capability to be seen in-person.” [36-year-old White man, Midwest] “to do a telemedicine visit with a psychiatrist, I had absolutely no place in my home away from my husband to have a private conversation…For me, my husband was—it's hard to believe that a well-educated woman, like me, I mean, I always vowed this would never happen, but I couldn’t even have my door closed to my room while I am on the phone or doing anything.” [73-year-old White woman, AZ]
Digital literacy			“First of all, I would’ve had to have my daughter come and help me if the appointment was online.” [82-year-old White woman, Arizona] “The barriers are we aren’t set up for it here at the house. I personally struggle with the technology probably more than somebody almost 73 should.” [72-year-old White man, FL] “I’m an older person, but people who are older than me have a problem using computers sometimes. It’s more a mental thing than anything. I think they don’t want to learn computers or do anything with them.” [66-year-old White woman, AZ]
Internet access			“I do not have internet, and as a matter of fact, I’m standing in the one place on my property where I have service right now.” [30-year-old White man, Midwest] “Yeah, ‘cause some places it may take them a while to link up, and by the time they send it, it’s dead.” [62-year-old African American man, Midwest] “The internet connection living in a rural area is a harder thing. We use a DSL line, so it is supposedly high-speed internet over a phone line, but it’s really not high speed, and a lot of the video sorts of things, the few times I did have to do Zoom calls for work or other social clubs, etcetera, part of my unsatisfaction with it was just that it was choppy, cutting out, etcetera, a lot.” [36-year-old White man, Midwest]
Body intimacy			“Well, yeah, there are a couple I would prefer face-to-face. One of them is urology…I would prefer that because I’ve had surgery in that area, and I would prefer talking face-to-face.” [84 -year-old African American man, AZ] “I really don’t want to point the camera in some places that you need to point in a doctor’s visit.” [62-year-old American Indian man, AZ]
Billing			“I don’t know that I would be comfortable getting charged the same amount if it was a telehealth appointment versus face-to-face.” [55-year-old White man, Midwest] “Well, what they need to do for billing is they need to know a hundred percent whether the insurance covers it or not before do it. The thing about it, I should not pay 240$ for something and come to find out my insurance paid for it.” [60-year-old African American man, AZ]
Patient portal difficulties			“Well, I guess it comes on where you have to put in your password or whatever. That’s where I’m stuck. I don’t know what I’m doing wrong. I just have trouble.” [82-year-old White woman, AZ] “If you’re depending entirely on a phone, it’s harder because it’s a little screen. Then you have to get into the app, you have to type it with two fingers. Not everybody is good with that.” [57-year-old Asian man, Midwest] “I had some confusion trying to find the link to get to Zoom.” [54-year-old White woman, Midwest] “It’s just that it’s that technology barrier, and there’s so many people on the wrong side of that barrier right now that I think the patient portal is just—it’s not—I don’t want to say useless, but it’s not the right way to handle the situation.” [30-year-old White man, Midwest] I just loathe the portal, and even if I type in emails I get back these cryptic answers from who knows who it is in the department, and I end up calling picking up the phone and waiting and waiting and waiting and discussing it…the information doesn’t make sense, and to me it’s more stressful than just waiting for my appointment...” [71-year-old White woman, FL] “I didn’t know that I needed to download Zoom, so that was a barrier before the appointment. It was not a seamless link from your portal.” [53-year-old White woman, FL]
**Recommendations for improving telehealth appointments**			
	Initial visits face-to-face; follow-ups on video	“It helps if you have one physical visit. For example, if you’re seeing somebody for the very first time, and you’ve never met them before, it might help that first visit is in-person...Because that way you’ve actually met. You’ve made eye contact once. Your comfort level has gone up a little bit. Now to conduct a second, third or follow-up calls on video might be a good idea.” [57-year-old Asian man, Midwest] “Maybe have the initial visits face-to-face and then subsequent visits over video.” [54-year-old White woman, Midwest] “...If I knew the provider, if I felt confident that the provider knew about the condition we were talking about, I think it would be idea for some follow ups.” [36-year-old White man, Midwest]	
	Recommendations to help those with low digital literacy or limited internet access	“Just making sure that their systems are not bloated or bogged down to the point that if you are talking to a person that has DSL internet or even dial-up, you get out into the really rural areas where you don’t have cables for traditional high-speed internet, and that’s still a common reality in Minnesota and Wisconsin.” [36-year-old White man, Midwest] “Maybe you guys do this already but make sure that-make people aware that they can do that [receive help setting up telehealth appointments], and that people do that all the time. There’s help available to help get you set up. Once they do one and it goes well, I’m sure they would do it again.” [66-year-old White woman, Midwest]	

#### F2F Rapport

One of the most frequently mentioned barriers to engaging in video appointments was what we have coined “f2f rapport” (ie, the feeling that the typical flow of a social encounter or empathetic connection with a patient’s provider is disrupted in a video appointment). Further, 4 subdivisions of this barrier were identified based on the way it was expressed: visual, personability, auditory, and therapeutic.

##### Visual

Many participants suggested that not *seeing* one’s provider properly negatively affects rapport. These participants tended to be older patients. On further probing, several participants elaborated that it was the *feeling* of visual connection that is being lost during the video appointments.

##### Personability

Several participants expressed that video appointments lacked the “personal touch” of an f2f appointment and emphasized a higher level of comfort with their provider that allowed them to share their concerns more readily. These themes tended to cross all types of visits (primary care and psychiatry).

##### Therapeutic

Across psychiatry and primary care appointments, several participants stressed that they considered an f2f appointment therapeutic and appreciated engaging in f2f interaction with their provider, especially due to the social isolation and loneliness brought on by the COVID-19 Pandemic.

##### Auditory

A relatively small number of participants conveyed the f2f barrier in auditory terms. This was approximately half as common as expressing this barrier in visual terms. Participants emphasized their preference to “talk” or “speak” with their doctor and hear them directly rather than through a computer or smart device speaker.

#### F2F Diagnostic and Therapeutic Advantages

Many participants believed that, although their medical concerns could be addressed virtually, seeing their provider f2f would confer a diagnostic and therapeutic benefit (ie, participants felt that care they received in person would be superior to that received virtually). Furthermore, several participants called attention to the fact that physical examinations often discover unanticipated issues that would not be discovered over video.

#### Habit

Several participants expressed that their preference for f2f appointments was due to habit, routine, or preference for familiarity (ie, because they were not used to video appointments, had always done f2f appointments, and therefore implicitly felt more comfortable in f2f appointments).

#### Privacy and Internet Security

Several participants expressed a wide variety of concerns relating to patient privacy that can be categorized along 2 lines: fear of who might be listening in on either end of a video appointment and fear of who otherwise might have access to the content of a video appointment. The latter concern typically related to patient concern for compromise of the security of their internet connection. This concern was expressed in both psychiatry and primary care appointments.

#### Digital Literacy

Another common barrier was a lack of digital literacy. Numerous participants expressed concern that their lack of skill in operating technology prevented them from feeling comfortable utilizing video appointments. Moreover, many participants who themselves felt comfortable with technology expressed concern that elderly family members would struggle. Participants who communicated low digital literacy were overwhelmingly elderly or rural residing. In addition, with only one exception, participants with low digital literacy did not feel comfortable with nor regularly use the patient portal—a prerequisite for using video appointments. Some elderly participants noted that they generally depend on a family member to help them use technology (ie, a digital navigator), mostly their children, and this person is not always present and available to help.

#### Internet Access

Internet access was primarily a barrier to engaging in video appointments if the participant was from a rural area. All participants who identified themselves as rural residing conveyed that internet access was a barrier to engaging in regular video appointments.

#### Bodily Intimacy

A few participants mentioned that they felt uncomfortable exposing certain parts of their body over video or in uploading photographs of private parts. Concerns of this kind were limited to the patients who scheduled appointments with their primary care physicians for urological, gynecological, and dermatological problems.

#### Billing

Several participants expressed concerns with how billing would be handled and conveyed and that this constituted a barrier to engaging in video appointments. The participants conveyed that uncertainty about co-pays and how much expense the insurance company would cover created a barrier to video appointments. Furthermore, a few participants expressed that they would be uncomfortable being charged the same amount for a video appointment, feeling they were being provided a lower level of service compared to an in-person visit.

#### Patient Portal

Participants were also asked about their experiences with the patient portal, because the ability to use the portal is required to participate in video appointments. Most participants used the portal and found it to be mostly user-friendly, though participants who expressed they had low digital literacy nearly all struggled with the portal or did not use it. A few participants had trouble accessing the teleconferencing application required for video appointments from the portal. Most concerns with the patient portal, however, were particular to single patients and not recurrent. These individual concerns were highly reminiscent of low digital literacy concerns.

### Patient Suggestions for Increasing Utilization of Video Appointments

Few participants had suggestions to offer. However, the recurrent theme among those who did was that video appointments were inappropriate for the first appointment with a new provider but acceptable for follow-up visits once rapport had already been established. A few participants noted the need for an institutional digital navigator who could immediately provide assistance if they are not able to connect to their provider virtually.

## Discussion

### Principal Findings

Our qualitative study provides information on personal and environmental factors that affected patients’ choice to not use video appointments during the COVID-19 pandemic within a large multistate institution for nonemergent and noninterventional outpatient clinical care. This preliminary study qualitatively explores patients’ telemedicine perceptions including barriers. Our findings will help inform the development of a large-scale survey that will guide future targeted interventions to increase the utilization and ease of use of telemedicine services.

The accumulated evidence from the early period of the COVID-19 pandemic (when this study was designed) showed that people had several misconceptions about the spread of COVID-19 [18] as a result of misinformation spread through social media platforms and other outlets. For example, some people believed COVID-19 was just a severe common cold without significant mortality [19]. In addition, before the development of vaccines, erroneous claims of achieving herd immunity by exposure to the virus were made [20]. Through the questions within domain 1 of our study, participants were prompted to talk freely about their COVID-19–related beliefs and perceptions and if those beliefs affected their decision to participate in f2f appointments. Our study is the first to explore such an association qualitatively. The results of our study indicate that the COVID-19 misconceptions may not have played a role in participants’ decisions to engage in f2f appointments. All participants in our study followed institutional and federal measures to mitigate the spread of COVID-19 during f2f clinical encounters, which probably motivated them to continue with f2f clinic care.

Within the second domain, we explored barriers associated with video appointments and factors that led participants to continue engaging in f2f appointments during the pandemic. We were interested if historical barriers to video appointments continued to exist during the pandemic. Like studies before the pandemic [5], our results indicate that participants who reported digital access and digital literacy as barriers to engaging in telemedicine were mainly older adults. Studies conducted before the pandemic highlighted that older adults struggled more with a poorly designed end user interface such as small text and computer screens and other digital skill tasks (scrolling down a menu tab, familiarity with a patient portal, or accessing a video conference link via the patient portal), which was supported by the participants in our study. These findings add to the existing body of literature and demonstrate that digital barriers (broadband access, digital literacy, and poor end user interface) continued to exist during the pandemic. Therefore, people with interrupted and limited digital access, such as those living in rural areas and those with limited digital literacy, such as older adults, may have no choice but to rely on traditional health care delivery methods such as f2f appointments. However, prepandemic studies have shown that older adults have demonstrated willingness to engage in technology and participate in telemedicine programs if the noted barriers are addressed [21,22]. Therefore, we propose a clinical practice change of assessing the digital competency of every patient so that digital solutions can then be applied based on the identified problem area. For example, people with no broadband access could be referred to a nearby free public hotspot or Wi-Fi access while also highlighting strategies to reduce privacy and data security concerns. We believe that those with limited digital literacy could be connected with institution digital navigator or support [23].

Lack of rapport has been highlighted in prior studies as a barrier to engaging in telemedicine care across different demographic subgroups [24]. Through the qualitative nature of our research, we present new findings by exploring this barrier in-depth with respect to patient sensory inputs and perceived therapeutic satisfaction with telemedicine care. This granular categorization reflects the diversity in which participants perceive lessened feelings of personability or connection with the clinician in video appointments. The most common subdivision of the “lack of rapport” category was the visual emphasis, followed by personability, then therapeutic, and finally auditory. We anticipate that these subcategories may not work in isolation and often interact with one another to define patient experience and perception. For example, patients who have trouble visualizing or hearing their clinician may report poor personal connection (personability). Future studies using quantitative approaches could explore how these subdivisions interact with one another.

More importantly, the unique nature of each subcategory, as highlighted in our results, has practical implications and suggests that attempts to alleviate the “lack of rapport” barrier cannot be one-size-fits-all. Accordingly, we recommend that digital access solutions be investigated to ameliorate rapport-related concerns. For example, digital navigators could provide and help patients use loaner devices with higher resolution screens, superior audio quality, or better internet connectivity to reduce latency and delays during video visits. Yang and colleagues [25] studied the implementation of loaner smartphone devices to patients who did not have smartphones; however, only 72% returned the loaned smartphone within a 30-day window. Future research is necessary to assess the feasibility of this approach for health care delivery. Together, these efforts could help reduce the awkwardness or degree of disruption to the flow of a conversation between patients and providers and increase the degree to which patients feel they can generate social rapport with their providers over video appointment.

Our results indicate that another common barrier to engaging in video appointments was perceived f2f therapeutic advantages. Participants believed their clinician would render better care in person (f2f) than over video. Participants pointed out that video appointments cannot replicate their perceived benefits of physical exams during f2f appointments. Assessing “lack of rapport” and “f2f therapeutic advantages” themes together, it can be inferred that patients perceive a video appointment as subpar (ie, of lower value either monetarily or therapeutically or diagnostically than an f2f interaction). Without providing exact reasons, several participants believed that psychiatry was a specialty well suited to video appointments. Participants could be inferring that psychiatry, unlike many other specialties, does not use physical exams to the same extent and thus would translate more effectively into a virtual format. The participants, however, did not elaborate if they would be comfortable discussing sensitive and personal topics in psychiatry via video.

On the contrary, participants were hesitant to engage in video appointments when they had dermatological, gynecological, or urological concerns. Several participants communicated that they needed to be examined in person and struggled with the possibility of using uploaded photographs of problem areas or exposing such sites to their webcams for providers to view. As highlighted by a few participants, this barrier could be due to concerns about privacy and security as well as awkwardness and discomfort relating to exposing more intimate body parts over video or uploading a photograph of the intimate body area. Although it seems probable that awkwardness and discomfort are the reasons driving the expression of this barrier, it is also possible the security or privacy barrier is at least compounding the concern if not occasionally replacing it. Moreover, concerns about security are potentially compounded by patient discomfort with the possibility, no matter how remote, of others gaining access to images of their bodies. Many patients expressed data privacy and security concerns when engaging in video visits, which has been highlighted as a barrier in the prepandemic literature [26]. Health care institutions need to vet their technological collaboration to assure patients by demonstrating the best practices regarding patient information privacy, data transfer, and storage [27]. A patient concerned about internet security for telemedicine from their end (user Wi-Fi or internet safety) could be connected to the digital navigator within the institution to assist patients.

### Limitations and Strengths

As this was a qualitative study, our findings cannot be generalized beyond our purposeful sample. However, we opted for a purposeful selection to ensure that we interviewed a diverse group of patients who would express a wide range of views. The study sample was from Mayo Clinic patients; therefore, the participant’s perception of a “safe environment” may not be transferred to other institutions. Additionally, we asked patients to recall a health care experience that had occurred almost a year prior. Hence, it is possible that their recollections and details about events could be biased or incorrect. To help ease such a concern, we did verify eligibility and the existence of an f2f appointment via the medical record. Even though we tried to enroll the participants from diverse backgrounds, most of the participants in our study had bachelor degrees and higher education, which adds to education bias.

Our study’s primary strength was the use of qualitative means to gain a thorough, rich, in-depth understanding of participants’ perceptions relating to barriers to health care and telemedicine during the COVID-19 pandemic. We underscore that such qualitative exploration is a necessary precursor to further investigation by more rigorous, quantitative means. As a next step, we plan to develop a quantitative survey using a larger and more representative sample to determine the extent to which our findings can be generalized

### Conclusion

Our study provides an in-depth investigation into barriers to engaging in video appointments for nonemergent clinical care. Limited f2f rapport, poor digital access and literacy, and concerns about privacy and security continued to be significant factors for patients not engaging in video appointments during the pandemic. Most importantly, this study highlighted that rapport-related concerns need to be looked at and problem-solved based on individual needs. Considerable clinical practice changes are required in the future at the institutional and policy level to encourage patients to engage in virtual care.

## Appendix

Multimedia Appendix 1 Interview guide.

## Abbreviations

f2f: face-to-face
RUCA: Rural-Urban Commuting Area
