# Supplementary material for: Patient Telemedicine Perceptions During the COVID-19 Pandemic Within a Multi-State Medical Institution: Qualitative Study
Source: JMIR Form Res. 2022 May 13;6(5):e37012. doi: 10.2196/37012 (PMC9109780; doi:10.2196/37012)
Supplement: Multimedia Appendix 1 [file formative_v6i5e37012_app1.docx]

Interview date: _____________________ Study ID: NSITE-1- ____ ____ ____

Type of visit:

- Psychiatry/psychology
- Primary care (Internal Medicine/Family Medicine)

Interview duration: ____ ____ minutes

**Q area 1: COVID-19 Health Experience and Access to Health Care**

**1. For this first question, we want to talk about the time of the pandemic before we had a vaccine, which would be about March/April 2020 all the way to about December 2020. Please tell me about what your life was like during this time.**

**PROBES (let interviewee talk, but include these questions if they aren’t addressed or the interviewee is having trouble remembering/getting started**

**Let’s start with the beginning of the pandemic…where were you living and who was with you? (i.e. alone with family, kids, parents); how did that change during the time of April-December 2020?**

**What was your work situation like? (i.e. were you working or furloughed laid off, what kind of work were you doing?); how did that change during the time of April-December 2020?**

**Can you talk about how the pandemic affected your mood and stress level (what was your mental health like?)**

**What was your physical health like during the pandemic?**

**How did the pandemic affect your healthcare? (i.e. can you talk about how the pandemic affected your decisions to seek or delay seeking care?)**

**Q Area 2: In Person Health Visit Experience and non-use of telehealth/video appointment options**

**1. Now, could you please describe to me what your experience was like when you received in-person care that was not an emergency at one of our clinics DURING the pandemic, all the way from how it was scheduled to what it was like going in?**

**PROBES (let interviewee talk, but include these questions if they aren’t addressed or they are having trouble remembering/getting started**

**What was your experience like when you got in contact with the clinic to get help figuring out what to do about your health concern? (i.e. what happened when you called to schedule an appointment?)**

**Who did you call (a nurse or a scheduler)?**

**What options were you offered to receive your healthcare (i.e. were you offered a video visit or to have your doctor visit you by video instead of in-person)?**

**How were safety concerns addressed during your in-person visit?**

**[If video visit options were offered but NOT chosen]: Why did you chose in-person care?**

**2. What kinds of concerns or barriers would you have to using video appointments that you might have?**

**PROBES (let interviewee talk, but include these questions if they aren’t addressed or they are having trouble remembering/getting started**

**How did (or might) your beliefs about how COVID-19 spreads affect the decision to use video appointments?**

**How did (or might) your trust in Mayo Clinic affect this decision to use video appointments?**

**How could concerns about billing affect this decision to use video appointments? (PROBE: insurance coverage)**

**How might your home life affect your ability to have a video appointment (PROBE: other people in the house, privacy)**

**How does (or might) your internet affect your ability to have a video appointment? (PROBE: Internet Access)**

**How does (or might) your ability to navigate computer, cellphone, internet (technology) have a video appointment? (PROBE: digital literacy)**

**How does your medical condition affect your ability to have a video appointment?**

**Can you tell us about how some of your concerns could have been handled by video?**

**How could some of your concerns would not have been easily handled by video?**

**What other kinds of concerns or barriers did you (or would you) have about video appointments during the pandemic?**

**3. In order to attend a video appointment, you have to have signed up for the Patient Portal which can be accessed either by a computer or a smartphone. Can you please talk to me about your experience(s) with the portal?**

**PROBES (let interviewee talk, but include these questions if they aren’t addressed or they are having trouble remembering/getting started**

**What kinds of services do you use your patient portal for? (scheduling appointments, messaging providers for medical concerns or medication refills)**

**What do you like about the patient portal?**

**What makes it difficult for you to use the patient portal?**

**How could we have made using the patient portal easier for you?**

**How could we make the patient portal easier for our patients who live in rural areas?**

**Q Area 3: Recommendations to increase use of video appointments**

**1. What could we have done to make it easier for you to schedule a video appointment?**

**2. What could we have done to make it easier for you to have the appointment by video?**

**3. Now I’d like you to think a minute on your decision to receive face to face care during the pandemic and share anything else about that decision that we haven’t yet already discussed?**

**Since December 2020, have you used video appointment(s)?**

- **Yes**
  - **Would you like to continue to use IF Mayo Clinic decides it as a** STANDARD future **rather than face-to-face appointments?**
    - **Yes (which specialty? What suggestions do you have to improve the experience?)**
    - **No (which specialty? what suggestion do you have to improve the experience?)**
  - **Would you like to continue to use video appointments if Mayo Clinic continues to offer this an OPTION for your care?**
    - **Yes (which specialty? What suggestions do you have to improve the experience?)**
    - **No (which specialty? what suggestion do you have to improve the experience?)**
- **No**
  - **Would you like to CONSIDER using IF Mayo Clinic decides it as a STANDARD in future rather than face-to-face appointments?**
    - **Yes (which specialty? What suggestions do you have to improve the experience?)**
    - **No (which specialty? what suggestion do you have to improve the experience?)**
  - **Would you like to CONSIDER using video appointments if Mayo Clinic continues to offer this OPTION for your care?**
    - **Yes (which specialty? What suggestions do you have to improve the experience?)**
    - **No (c**

**End of interview**

End Time: ___ ___: ___ ___ I have ended the recording. Did you have any questions about the interview portion?

**Survey Questions:**

**Now, I am going to ask you a few survey questions:**

**The following questions are asking you to rate on you felt the following measures for slowing the spread of COVID-19. On a scale from 1-4, with 1 being extremely ineffective to 4 being extremely effective.**

**Thinking back on when the COVID-19 pandemic first started (from March/April to about December 2020) what were your beliefs about the following actions for stopping COVID-19?**

| How effective was… | 1 Extremely Ineffective | 2  Somewhat Ineffective | 3  Somewhat Effective | 4  Extremely Effective | 5  Unsure |
| --- | --- | --- | --- | --- | --- |
| Wearing a facemask |  |  |  |  |  |
| Social distancing (6ft distancing) |  |  |  |  |  |
| Washing and sanitizing your hands |  |  |  |  |  |

**Thinking back on when the COVID-19 pandemic first started (from March to about December 2020)**

**Were you or anyone in your household diagnosed with COVID-19? (Select all that apply)**

- **Yes**
  - **If Yes, who?**
    - **Self**
      - I am recovering or recovered at home
      - I was admitted to the hospital
      - I was in the ICU
    - **Household (specify)_______**
      - They are recovering or recovered at home
      - They are at or went to the hospital for COVID-19
      - They are at or went to the ICU for COVID-19
      - They died from COVID-19
- **No***

**PERCEIVED RISK**

- 5-Doesn't apply: participant had COVID-19

Thinking back on when the COVID-19 pandemic first started (from March to about December 2020)

**On a scale from 1-4, with 1 being not at all likely to 4 being very likely,** How likely did you think it was that you could get COVID-19?

- 1-Not at all likely
- 2-Somewhat likely X
- 3-Likely
- 4-Very Likely

**PERCEIVED SEVERITY**

- 5-Doesn't apply: participant had COVID-19

Thinking back on when the COVID-19 pandemic first started (from March to about December 2020),

**On a scale from 1-4, with 1 being not at all serious to 4 being very serious,** If you got COVID-19, how serious did you think it would have been for you?

- 1-Not at all serious X
- 2-Somewhat serious
- 3-Serious
- 4-Very serious

**About You**

**RACE/ETHNICITY**

What race are you?

- White/Caucasian
- Hispanic Latinx
- Black/African American
- American Indian or Alaska Native
- Asian
- Native Hawaiian or Pacific Islander
- I prefer not to share
- Don’t know
- Other
  - If other, please specify
- 2 or more
  - If 2 or more, please specify

**GENDER IDENTITY**

Which gender do you identify with?

- Female
- Male
- Nonbinary or Genderqueer
- Transgender Female/male-to-female
- Transgender Male/female-to-male
- I prefer not to share
- Don’t know
- Other
  - If other, please specify

**RELATIONSHIP STATUS**

What is your current relationship status?

- Single
- Committed Relationship
- Engaged
- Married
- Life Partnership
- Separated
- Divorced
- Widowed
- I prefer not to share
- Don’t know
- Other
  - If other, please specify

**EDUCATION**

What is the highest level of education you completed?

- I didn’t go to school
- Up to 8^th^ grade
- Some high school
- High school graduate / GED
- Trade / technical school
- Some college credit, no degree
- Associate’s degree
- Bachelor’s degree
- Graduate degree
- I prefer not to share
- Other
  - If other, please specify

**POLITICS**

In politics today, what do you consider yourself to be?

- Democrat
- Independent
- Republican
- I prefer not to share
- Don’t know
- None
- Other
  - If other, please specify

**RELIGION**

In religion today, what do you consider yourself to be?

- Hinduism
- Buddhism
- Islam
- Confucianism
- Christianity
- Taoism
- Judaism
- Atheist
- Agnostics
- I prefer not to share
- Don’t know
- None
- Other
  - If other, please specify

That is all I have for questions. Thank you for your time. We appreciate your taking part in this interview. Your thoughts are greatly appreciated and very helpful.

To confirm we have the correct address for you, please confirm what I have on file for you (list what is in Epic/Ptrax).
